# The utility of ‘home-made’ reagent red blood cells for antibody screening during pre-transfusion compatibility testing in Uganda

**DOI:** 10.4314/ahs.v21i2.38

**Published:** 2021-06

**Authors:** Bernard Natukunda, Robert Wagubi, Ivan Taremwa, Benson Okongo, Yona Mbalibulha, Gayle Teramura, Meghan Delaney

**Affiliations:** 1 Division of Hematology and Transfusion Medicine, Faculty of Medicine, Mbarara University of Science and Technology, P. O. Box 1410, Mbarara, Uganda; 2 Department of Internal Medicine, Mbarara Regional Referral Hospital, P. O. Box 40, Mbarara, Uganda; 3 Institute of Allied Health Sciences, Clarke International University, P. O. Box 7782, Kampala, Uganda; 4 Department of Medical Laboratory Sciences, Faculty of Medicine, Mbarara University of Science and Technology, P. O. Box 1410, Mbarara, Uganda; 5 Bloodworks Northwest Immunohematology and RBC Genomics Reference Laboratory, 921 Terry Avenue, Seattle, Washington, 98104, United States; 6 Department of Pathology and Laboratory Medicine, Children's National Health System, 111 Michigan Avenue NW, Washington DC, 20010, United States

**Keywords:** Blood transfusion, ‘Home-made’ reagent RBCs, Pre-transfusion testing, RBC alloantibody screening, Uganda

## Abstract

**Background:**

The WHO recommends that pre-transfusion testing should include ABO/RhD grouping followed by screening for red blood cell (RBC) alloantibodies using the indirect antiglobulin test (IAT). However, in Uganda, current practice does not include RBC alloantibody screening.

**Objective:**

To assess the utility of ‘home-made’ reagent RBCs in alloantibody screening.

**Materials and Methods:**

In a laboratory-based study, group O RhD positive volunteer donors were recruited and their extended phenotype performed for C, c, E, e, K, Fya, Fyb Jkb, S and s antigens. These ‘home-made’ reagent RBCs were preserved using Alsever's solution and alloantibody detection tests performed. For quality assurance, repeat alloantibody screening of patients' samples was done at Bloodworks Northwest Laboratory in Seattle, United States.

**Results:**

A total of 36 group O RhD positive individuals were recruited as reagent RBC donors (median age, 25 years; range, 21 – 58 years; male-to-female ratio, 1.6:1). Out of the 311 IATs performed, 32 (10.3%) were positive. Confirmatory IAT testing in the United States was in agreement with the findings in Uganda.

**Conclusion:**

Use of ‘home-made’ reagent RBCs during pre-transfusion testing in Uganda is feasible. We recommend the introduction of pre-transfusion IAT alloantibody screening in Uganda using ‘home-made’ reagent RBCs to improve transfusion safety.

## Introduction

According to the World Health Organization (WHO), pre-transfusion testing procedures should include ABO/RhD blood grouping followed by the indirect antiglobulin test (IAT) screening for red blood cell (RBC) alloantibodies that could cause hemolysis in the transfusion recipient. A direct test of compatibility (the cross-match) is then performed before the blood is infused^1^. However, in Uganda current practice is limited to ABO/RhD typing plus room temperature (RT) saline cross-matches without additional 37°C and IAT testing. Pre-transfusion screening for immune RBC alloantibodies among blood recipients is not performed even in cases with a history of previous transfusion or pregnancy due to the lack of the necessary reagents and supplies. However, it has been reported that one out of every 16 (6.1%) previously transfused patients in Uganda possesses clinically significant RBC alloantibodies, suggesting that there is a compelling medical rationale to perform IAT testing prior to transfusion 2, 3.

RBC alloimmunization usually occurs due to the genetic differences between the blood donor and the recipient on the one hand and the pregnant woman and the fetus on the other. Transfusion-acquired antibodies have been implicated in immediate and delayed hemolytic transfusion reactions (HTRs)^4^; some patients with multiple antibodies are ‘difficult to transfuse’ and others develop autoantibodies in addition to being alloimmunized^5^,^6^. Furthermore, RBC alloimmunization may limit the availability of compatible blood for future transfusions and can contribute to perinatal morbidity and mortality consequent to hemolytic disease of the fetus and newborn (HDFN)^7^. In well-resourced settings, post-transfusion RBC alloimmunization is prevented by phenotypic matching for C, E and K antigens before the start of a transfusion program for multiply transfused patients such as those with sickle cell disease^8^,^9^. Pre-transfusion screening for RBC alloantibodies helps in alloimmunization detection in order to prevent the occurrence of acute and delayed HTRs. When pre-transfusion testing detects the presence of RBC alloantibodies, these are first identified so that antigen negative and compatible units of blood are transfused. Although previous reports indicated that the introduction of pre-transfusion RBC alloantibody screening in Uganda would be cost-effective^10^, there have been concerns on the additional costs involved and the lack of funds for the procurement of commercial reagent RBC supplies obtained from overseas since there are no African manufacturers of these specialized reagents. In contrast, in high index settings reagent RBC panels are sourced, packaged and prepared by commercial manufacturers and then supplied to hospital blood banks for use in testing patient samples. Furthermore, even if these reagents were financially accessible to low income countries, they are temperature sensitive and may not remain stable unless there is strict maintenance of the cold chain during long distance shipment and transportation to final destinations. In Uganda, the local hospital transfusion laboratories are as understaffed as they are ill-equipped and hence unable to handle most of the gel and microplate-based testing platforms that are currently available on the market. A more plausible solution to this challenge is the use of the traditional tube test and ‘home-made’ reagent RBCs from local blood group O RhD positive volunteer donors at regional blood centers of the Uganda Blood Transfusion Service (UBTS). RBC alloantibody screening on patients can then be performed using the equipment available at hospital laboratories such as microscopes, centrifuges, water baths, test tubes and refrigerators. Therefore, the aim of this study was to assess the utility of ‘home-made’ reagent RBCs in the implementation of pre-transfusion RBC antibody screening in the Ugandan setting.

## Methods

In a laboratory-based study, blood group O RhD positive volunteers that were either staff or students at Mbarara University of Science and Technology (MUST) in Mbarara, Uganda, were mobilized and recruited as reagent RBC donors. The study took place between May and August 2017, in the Blood Transfusion Laboratory at Mbarara Regional Referral Hospital (MRRH) in Mbarara, Uganda. Approval to conduct the study was sought from the Research and Ethics Committees at MUST and clearance was received from the Uganda National Council for Science and Technology (UNCST). Informed consent was obtained from the volunteer donors and their demographic details were recorded. Eligible RBC donors were group O RhD positive individuals who had no history of blood transfusion or pregnancy. However, the volunteer donors were not screened for transfusion transmissible infections (TTIs) including HIV, HBV and HCV.

Fifteen milliliters (15mL) of venous whole blood were collected from each volunteer into anticoagulated Falcon tubes (Corning). Of this, 4mL were put into ethylenediaminetetraacetic acid (EDTA) vacutainer tubes for ABO/RhD grouping and extended phenotyping for the following RBC antigens: C, c, E, e, K, Fya, Fyb, Jkb, S and s using commercial antisera (Ortho Clinical Diagnostics) that were kindly donated by Bloodworks Northwest in Seattle, United States. The remaining blood in the Falcon tubes was used for preparation of the ‘home-made’ reagent RBCs. Reagent cell preparation requires phenotyping for multiple RBC antigens in order to characterize the extended blood type. A 2 - 5% saline suspension of RBCs from the volunteer donors was prepared and mixed with each of anti-C, anti-c, anti-D, anti-E, anti-e, anti-K, anti-Jkb and anti-S in separate test tubes (serum-to-cell ratio, 2:1). The mixture was incubated at RT for 10 minutes (tube test) and the agglutination results were interpreted according to standard procedures to determine if the reagent cells had the corresponding RBC antigens. Positive and negative controls for each of the antisera were not available and the test results were not verified by a second technologist. For the anti-Fya, anti-Fyb and anti-s antisera, an IAT phase was also performed according to the manufacturer's instructions (Ortho Clinical Diagnostics). Anti-human globulin (Coombs') reagent containing anti-IgG (Immucor Inc.) and IgG sensitized RBCs (Coombs' control cells) were used in the IAT technique. The extended phenotypes for each RBC antigen of each volunteer donor were interpreted and recorded. The fully characterized reagent RBCs were dispensed into 10mL vials under aseptic conditions. The reagent RBCs were labelled with batch numbers as well as the date of preparation. Basing on the antigenic profiles of the donors, reagent RBCs were arranged into sets of 3-cell panels to have the right antigens positive with each panel containing at least one RBC vial positive for each of the antigens tested. The reagents RBCs were preserved using Alsever's solution (Sigma-Aldrich) and kept refrigerated at 2 – 8°C for up to a 4-week period. However, there was no repeat testing of the patient plasma using the same reagent RBCs at the end of the above storage duration. Also, no repeat phenotyping of theRBC panel cells was performed at the end of the 4-week period due to limited quantities of the typing antisera available. In all cases, a Tabletop centrifuge (Hettich); a water bath (Memmert); and 12 x 75mm Kahn tubes were used.

The ‘home-made’ reagent RBCs were then used for preand post-transfusion antibody screening of patients who were participants in the parent IPAT study, whose findings were published elsewhere12. As a quality control check, repeat IAT testing (using the tube method) of all positive patient samples using commercial RBC panels was carried out in the United States at the Immunohematology Reference Laboratory of Bloodworks Northwest, Seattle, WA.

## Results

A total of 36 blood group O RhD positive individuals; with 14 of them being female (median age, 25 years; range, 21 – 58 years; male-to-female ratio, 1.6:1) were recruited as volunteer donors for the ‘home-made’ reagent RBCs. Over a 4 month period, at the beginning of each month 9 volunteer donors were bled giving a total of 12 batches of 3-cell panels for use during the study. The reagent cells had their extended phenotype performed to characterize the RBC blood group system antigens involved (Table 1).

**Table 1:** Phenotyping results for the group O D positive reagent RBC volunteer donors (n=36)

Blood group	C	c	E	e	K	k*	Fy^a^	Fy^b^	Jk^a^*	Jk^b^	S	s
Positive (%)	19	96	22	94	3	NA	3	5	NA	11	33	44
Negative (%)	81	4	78	6	97	NA	97	95	NA	89	67	56

* NA=Not applicable.

*Note:* Among the antisera kindly donated from Bloodworks NW Laboratory in Seattle, USA, anti-k and anti-Jk(a) were not provided.

The ‘home-made’ reagent RBCs were used to screen 311 samples from previously transfused patients (110 pre-transfusion and 201 post-transfusion screens) employing the IAT technique. Using these reagent cells, 32 (10.3%) out of the 311 IATs were positive. Overall, there were 27 patients found to be alloimmunized to RBC antigens at the time of follow up with a rate of post-transfusion RBC alloimmunization of 12.3%. Confirmatory studies performed in the United States on samples from the patients were in agreement with the above serological findings.

## Discussion

A major reason why the WHO recommendations on pre-transfusion testing are not yet implemented in Uganda is the lack of finances for the procurement of specialized blood banking reagents needed for the procedure. This “proof of principle” project was undertaken at Mbarara Regional Referral Hospital in Mbarara, Uganda. It was part of the larger IPAT study^12^, a randomized controlled trial that assessed the role of improved pre-transfusion testing in the prevention of delayed transfusion reactions among transfused patients in Uganda. Eligible participants in the IPAT study had a history of prior RBC exposure through blood transfusion and/or pregnancy. Due to the significant post-transfusion RBC alloimmunization previously observed among Ugandans, it was recommended that there was a need to introduce RBC alloantibody screening tests in local hospital blood banks on patients before receipt of transfusions^3^. Given the lack of funds for the monthly procurement of commercial reagent RBC panels from foreign manufacturers, we assessed the utility of ‘home-made’ screening cells from local volunteer blood donors as an alternative.

This concept of ‘home-made’ reagent RBCs was informed by research reports on the use of “laboratory made” RBCs for reverse ABO grouping by investigators in low budget settings in Algeria and Madagascar^13^,^14^. The innovation is based on the blood banking technology developed in the 20^th^ Century and will be beneficial for Uganda and other resource-limited countries in sub Saharan Africa where pr-transfusion testing practices are still substandard due to financial constraints. In Uganda, patients are at an increased likelihood of getting transfusion reactions because the crucial intermediate step of 37°C IAT screening for RBC alloantibodies is not carried out, putting them at high risk for immunohemolytic complications. As a preventive measure, we hereby advocate for a new policy in Uganda giving rise to the introduction of screening for RBC antibodies during pre-transfusion testing. Once introduced in Uganda, RBC alloantibody screening procedures can support transfusion safety and lead to better post-transfusion outcomes by way of averting the consequences of RBC alloimmunization, including HTRs and HDFN. By using ‘home-made’ reagent RBCs, there will be a substantial reduction in costs. For instance, a 10 mL vial of reagent RBCs can be used to screen about 200 patients – using the tube test – and the cost of a set of a 3-cell panel of reagent RBCs (imported from manufacturers overseas) for pre-transfusion testing is about 150 USD which is equivalent to approximately 2.0 USD per blood recipient screened^10^. Hence, this study has demonstrated that the strategy of using ‘home-made’ reagent cells is a more feasible and cheaper alternative compared to the imported reagent RBC panels. In this study, cases with a positive pre-transfusion RBC antibody screen were transfused with compatible units of blood following 37°C IAT cross-matches. Importantly, repeat screening of the patient samples performed using commercial reagent cells (tube test) in the United States yielded concordant findings with no major discrepancies observed save for slight variations in the reaction strengths.

While the use of ‘home-made’ reagent RBCs was found to be feasible, our research project had some limitations. Volunteer donors were not screened for TTIs and the reagent RBCs were not phenotyped for Jka, k, M and N antigens. There was a possibility that some of the negative antibody screens could have actually been positive given a different antigen mix in the reagent cells. Due to study constraints, an assessment of all antibody screen negative results could not be confirmed using various antigen mixes. Blood group genotype analysis was not performed to confirm whether the RBC antigens had a variant expression, which would normally be done on commercially available reagent RBCs. This has implications on the strength and sensitivity of antigen-antibody reactions observed especially for RBC antigens that usually show dosage e.g. those in the Rh and Duffy blood group systems^15^. Also, there was no opportunity to assess the utility of the ‘home-made’ reagent RBCs in alloantibody identification procedures since these techniques were beyond the scope of the local staff and available resources.

Notwithstanding the above limitations, the following recommendations can be made as a way forward. The UBTS, which is the national blood supplier, could be involved in the roll out program for the ‘home-made’ reagent RBCs because there will be access to a large number of potential reagent cell donors. Therefore, a combination of RBCs expressing the right antigens positive could be provided in each 3-cell panel corresponding to the common clinically significant antibodies. The regional blood centers of the UBTS could mobilize the volunteer donors, screen them for TTIs, and perform extended phenotyping for their RBC antigens including those for Jka, k, M and N antigens. Since the reagent RBC donors will be locally available within the communities, they could easily be traced and identified by the UBTS in case repeat samples are required. Thereafter, the ‘homemade’ reagents could be supplied together with blood and blood components to hospitals and other transfusing health facilities. Eligible and consenting individuals will be transferred from the general donor inventory to become reagent RBC donors who can be called upon to donate as necessary but usually about once every 3–4 months as recommended^16^. In liaison with other stakeholders, the UBTS could formulate standard guidelines on the “manufacture” of these ‘home-made’ reagent RBCs, and address other quality issues such as the use of positive and negative controls as well as confirmatory testing by a second laboratory technologist. The reagent RBCs will be prepared according to specifications within a quality-controlled environment before being supplied to corresponding blood banks within the vicinity. Anti-human globulin (Coombs') reagent and sera for extended phenotyping could be procured by the National Medical Stores (NMS) alongside drugs and sundries.

Laboratory and clinical staff will be sensitized and trained on the significance of this new pre-transfusion testing procedure. It is recommended that additional 37°C IAT cross-matches should be performed for recipients with negative antibody screens using the ‘home-made’ reagent cells because these panels may not include RBCs possessing antigens - preferably in a homozygous manner - corresponding to the common RBC alloantibodies. Clinical review groups or hospital transfusion committees will be formed at all transfusing health facilities to help in the monitoring and evaluation of blood transfusion outcomes following this intervention. Fortunately, the ‘home-made’ reagent cells can also be used in antenatal screening for maternal RBC alloimmunization since this is currently not part of routine obstetric care in Uganda despite a reported anti-D alloimmunization rate ranging from 6 -12% among pregnant women in the country^17^, ^18^. There is potential for an extra role of these reagent RBCs for alloantibody identification purposes once the necessary laboratory infrastructure is in place and antigenic profiling of our volunteer donors has been carried out using molecular immunohematologic techniques.

## Conclusion

We strongly advocate for an improvement in pre-transfusion testing in Uganda by introducing the use of ‘home-made’ reagent RBCs for antibody screening. Categories of patients that will mostly benefit from the additional serologic testing procedures include sickle cell disease (SCD) and other multiply transfused (OMT) individuals because, from previously published scientific studies, they are known to have a high risk for RBC alloimmunization and the consequences thereof.

## Abbreviations

IAT: Indirect antiglobulin test; IPAT: Improved pre-transfusion testing to prevent anamnestic serologic reactions following transfusions; RBC: Red blood cell; RT: Room temperature; USD: United States Dollars; WHO: World Health Organization.
